# Reveal the dynamics of mobile health services continuance intention: effects of expectation, confirmation, and chronic disease

**DOI:** 10.3389/fpubh.2025.1637264

**Published:** 2025-09-23

**Authors:** Xiumei Ma, Yanxia Li, Ao Suo

**Affiliations:** ^1^School of Public Administration, Sichuan University, Chengdu, China; ^2^School of Economics and Management, Yanshan University, Qinhuangdao, China; ^3^Clinical Medical School, Gansu Medical College, Pingliang, China

**Keywords:** mHealth services, continuous use, latent growth model, expectation-confirmation model, chronic disease

## Abstract

**Introduction:**

The sustainable development of mobile health (mHealth) services relies on the continuous use by users. Most studies consider users’ intention to continue using mHealth services as a static measure rather than one that changes dynamically over time, often neglecting the impact of individual differences, such as the presence of chronic disease, on usage patterns.

**Methods:**

Drawing on the expectation-confirmation model, this study investigates the dynamic nature of the intention to continue using mHealth services, with a particular focus on the role of chronic disease. Conducting a longitudinal study with three rounds of online survey, we analyzed data collected from 236 completed respondents using a latent growth model.

**Results:**

The results indicate that users’ intention to continue using mHealth services is not static and tends to decrease over time. Expectation accelerates the descent, while confirmation mitigates it. Furthermore, expectation shows a stronger impact on users without chronic disease compared to those with chronic conditions.

**Discussion:**

This study advances the understanding of the continuous use of mHealth services by incorporating time-based dynamics and the influence of chronic disease. This study also extends the traditional expectation-confirmation model into a dynamic framework.

## 1 Introduction

Mobile health (mHealth) services have flourished in recent decades due to advancements in mobile communications and network technologies (1, 2). Compared to earlier electronic health services accessed via desktop computers (3–5), mHealth offers a more convenient way for patients to obtain remote health consultation, health monitoring, and customized information services (6). As an innovative approach to delivering health services, mHealth has been empirically proven to be effective in promoting health management, particularly by enabling patients with chronic disease to actively engage in self-care (7). Due to these advantages and conveniences, mHealth services have garnered increasing attention and widespread popularity in recent years.

Although a growing number of studies have made initial efforts to investigate continuance issues within mHealth, most have viewed this phenomenon as static rather than dynamic (8). This is evident as many existing studies on mHealth continuance rely on cross-sectional surveys conducted at a single point in time (9, 10). However, users’ beliefs and use intentions are dynamic and vary over time (11). Initially, users may have a high intention to continue using mHealth services due to high expectations. However, the intention is likely to decrease or even disappear if the service performance does not meet their expectations. Although a few studies have emphasized the dynamic nature of mHealth continuance intention (12, 13), they have not empirically explored the change trajectory. Thus, incorporating a time dimension into empirical studies that examine changes in users’ intention to continue using will address this research gap, providing insights into users’ engagement, and promote the sustainable development of mHealth. Therefore, we propose the first research question:

RQ1: How does users’ intention to continue using mHealth services change over time?

The expectation-confirmation model (ECM) is widely used to understand individuals’ continuous use behavior and intention (14, 15). The original ECM posits that users’ expectation and confirmation influence satisfaction, which further leads to behavior or intention (16). Building on the ECM, Bhattacherjee and Premkumar (17) verified temporal pattern of technology usage and investigated how expectation and confirmation at different stages influence continuous use intention through longitudinal testing. However, they only emphasized that users’ beliefs and attitudes vary over time, failing to reveal the trajectory of change, especially how expectation and confirmation affect the change trajectory. Thus, while an expectation-confirmation paradigm is suggested to explain the continuous use of technologies, no study to date has examined how expectation and confirmation affect the change trajectory of continuous use intention. To fill this gap, the second research question is proposed:

RQ2: How do expectation and confirmation influence the change in users’ intention to continue using mHealth services?

Moreover, individual differences may also influence the continuous use of mHealth services. Research has indicated that individual traits such as gender (18), professional seniority (19), income (20), and health condition (21) interact with technology to shape user behavior. Specifically, individuals’ vulnerability to chronic disease has a contingent effect on mHealth use (22). This maybe because a fundamental component of mHealth services is assisting in chronic disease management. Thus, the demand for and use of mHealth services may differ between individuals with and without chronic disease. We are particularly interested in whether chronic disease involvement affects the change trajectory of mHealth continuous use over time. Specifically, we aim to understand how chronic disease interacts with users’ expectation and confirmation, as its role remains unclear. Therefore, the third research question of this study is:

RQ3: What role does chronic disease play in the change of users’ intention to continue using mHealth services?

To answer these three questions, we conducted a longitudinal test through three successive surveys. Utilizing the latent growth model, this study explores how use intention evolves over time, how expectation and confirmation influence the change trajectory, and the role of chronic disease in this process. This study sheds light on current literature and practice. First, this study views continuous use intention as dynamic and clarifies how it changes over time in the mHealth context. Given that most previous studies have treated individual intention to use as static (23, 24), this study expands the literature by diagnosing time-based dynamics of use intention. Second, this study provides a new understanding of changes in continuous use intention from the lens of expectation-confirmation paradigm. In particular, this work is among the first to combine the ECM with the trajectory of continuous use intention, presenting a clear picture of the impact of expectation and confirmation on the evolution of mHealth continuous use intention. Third, through examining the role of chronic disease, this study expands the literature on the ECM by exploring its applicability in contexts involving both presence and absence of chronic disease. Fourth, this study offers methodological guidance for future longitudinal studies. Finally, results in this study may inspire further research into the impact of chronic disease in the mHealth context and provide practitioners with actionable strategies for retaining mHealth users, both with and without chronic disease.

## 2 Theoretical background and hypothesis development

### 2.1 The dynamics of mHealth services continuous use intention

Continuous use is defined as the behavioral patterns reflecting sustained use of a technology by individuals over long time after adoption (25), which has attracted extensive attention in information systems and social science field (26). Continuous use of mHealth can not only ensure the economic return of an mHealth business but also improve the users’ health management. Most research about the continuous use of mHealth services have investigated determinant factors influencing users’ continuance intention from viewpoints of system features and user perceptions, seperately or concurrently (27–29). For example, drawing on the technology acceptance model and investment model, Cho et al. (30) investigated the role of perceived usefulness, perceived ease of use, and investment size in predicting mHealth continuance intention (67). Showed that individuals’ continuous intention was linked to service assurance, hedonic benefits, efficiency, reliability, and content quality. Such studies in the mHealth services context have generally viewed continuous use as unchanging or static, failing to understand how continuous use changes over time and explore the trajectory or the rate of the change.

However, continuous use is far from static: it is a dynamic process in which users may stop and readopt the technology, and studies as early as Bhattacherjee (31) have emphasized its dynamic aspects and nature. More recent studies have shown the importance of investigating the dynamics of continuous use in the mHealth context. For example, conducting a longitudinal study, Meyer et al. (32) discovered that users often break and abandon their mHealth devices during long-term use. Shen et al. (13) indicated that since users may behave intermittently, continuous use is not a fixed decision but a dynamic process. Thus, we argue that mHealth services continuous use intention decreases over time. This would align with one previous longitudinal study on technology use, in which use intention in a later stage was found to be lower than in an earlier stage (32). We therefore propose that:

*H1*: Individuals’ continuous use intention is dynamic and decreases over time.

### 2.2 Expectation confirmation model

Bhattacherjee (31) proposed the expectation confirmation model (ECM), a postadoption model addressing that individuals’ continuous use of information technologies or services. The ECM mainly focuses on the post-adoption variables and posits that confirmation of the initial expectation influences perceived performance and then influence users’ satisfaction and intention to continue using. In recent years, more scholars paid notice to ECM and applied this model in mHealth services continuance research (30, 33, 34).

However, despite the valuable theoretical insights the ECM has provided regarding continuous use, several research blanks remain. First, the ECM has widely been drawn upon to explain users’ continuous use behavior and intention related to a technology or service, but it has never been revoiced to address the dynamic development of continuous behavior and intention (35, 36). Given that some more recent studies have been stressing the importance of understanding how continuous use behavior and intention change during the whole use process (13), the ECM needs to be extended through considering the dynamic change of continuous use. Second, most current studies drawing on the ECM only focus on post-adoption variables (e.g., perceived performance, confirmation, satisfaction, and continuous use), while they have lost sight of pre-adoption variables (e.g., expectation) (37–39). Recent research has alerted researchers to investigate the whole adoption process, including the ways in which pre-adoption expectations shape post-adoption perceptions/behaviors (40, 41). Thus, in order to understand the whole use process, especially the initial use intention and changes in intention in the long-term use stage (e.g., the rate of change), it is critical to involve expectation and confirmation in the model.

Expectation refers to a user’s anticipated or predicted belief about the attributes or characteristics of technologies or services (42). When users believe technologies or services will perform well in the future, they are more likely to use them. The unified theory of acceptance and use of technology has also asserted that the initial usage intention is affected by the user’s expectations about the technology’s performance (43). That is, high-level expectation will increase user’s continuous intention to use mHealth services. In addition to influencing the initial use intention, expectation may have a significant effect on subsequent use, such as changes in continuous intention. Expectation, as users’ pre-use belief, serves as an anchor or a baseline for evaluating the technology or service. A higher baseline expectation is harder to satisfy, and thus users are more likely to incur a psychological loss during the subsequent use process. According to the theory of risk aversion, individuals will take measures to avoid risk, especially when risk aversion increases with time (44, 45). Specific to this context, high-level expectation is likely to bring a risk of psychological loss, and in response to this loss, users may reduce their continuous intention. Thus, we infer that the higher the initial expectations, the faster the continuous intention will decrease. Therefore, we propose that:

*H2*: Expectation is positively related to the initial continuous use intention.

*H3*: Expectation is positively related to the rate of change in continuous use intention.

Confirmation refers to users’ perceptions of the congruity between their expectations of a technology’s performance and its real performance (31). In this regard, confirmation is an outcome of comparing a user’s pre-adoption expectation and post-adoption perceived performance. A high-level confirmation indicates that technologies’ or services’ performance satisfies the user’s expectations, which should positively influence the user’s intention to use them, at least initially (31). Studies have demonstrated that confirmation significantly improves users’ attitude towards technologies and services as well as their intentions to continue using technologies and services (37, 40). In this study, we expect use intention to decrease over time during the use process (H1), perhaps substantially due to fatigue or tiredness. In this regard, confirmation may play a role in the change of use intention. Individuals who get high confirmation, and therefore high satisfaction, may experience a drop in use intention, though not as much or as quickly as other users. Individuals who get low confirmation (and low satisfaction) should see a faster decrease in their use intention. Thus, we put forward that:

*H4*: Confirmation is negatively related to the rate of change in continuous use intention.

### 2.3 The role of chronic disease

Chronic disease is often incurable and requires long-term management, which may include addressing diet and exercise behaviors, monitoring and recording real-time health data, and asking for medication advice from physicians (46). Chronic disease degrades individuals’ overall health condition and quality of life, so it negatively affects them both physiologically and psychologically (47). Physiological effects include poor health and drug dependence, and psychological effects include gloom and panic caused by poor health condition and psychological dependence on physician support. Due to these negative impacts, there are significant differences in health service needs between individuals with chronic disease and those without chronic disease (48).

Although mHealth can offer health management services and information to both chronic patients and individuals with no chronic disease, these two groups may have different demands and expectations regarding the function and performance of mHealth. The expectation and confirmation paradigm has been widely examined in mHealth services studies (41); however, the role of chronic disease has rarely been investigated. According to the expectation confirmation theory, expectation negatively correlates to confirmation since a higher expectation is harder to meet (49). Compared to individuals with no chronic disease, chronic patients have more and urgent needs as well as higher requirements for mHealth services to solve their problems (50). In this situation, their expectation is harder to satisfy and confirm than others’. Thus, expectation may lead to lower confirmation for chronic patients.

Moreover, the differing expectations of individuals with and without chronic disease may have differing impacts on their initial mHealth services use. For example, chronic patients tend to expect mHealth to perform well in solving their health problems, while individuals with no chronic disease are likely to use mHealth to learn more about their health conditions. Chronic patients using mHealth services may therefore place more emphasis on their performance expectations, while in contrast, users with no chronic disease may pay more attention to their interaction with the technology (22). In other words, chronic patients have higher use intention when they have high expectation while users with no chronic disease have higher use intention when they experience valuable interaction. That is, the impact of expectation before using mHealth is stronger for chronic patients. Thus, it is reasonable to argue that compared to individuals with no chronic disease, mHealth services expectation may play a more important role in motivating initial continuous use intention for chronic patients. Hence, we put forward that:

*H5*: The relationship between expectation and confirmation is stronger for chronic patients.

*H6*: The relationship between expectation and initial continuous use intention is stronger for chronic patients.

According to Maslow’s hierarchy of needs, protecting ourselves from disease and staying healthy is the basic and pressing need driving individuals to take action (51). For a chronic patient, using mHealth services is an efficient way to fulfill this need by managing their disease, and it has significant potential to improve their health. Thus, chronic patients tend to have more pressing mHealth needs. According to demand theory, more demand can cause a higher level of preferences and more quantity of services that individuals would like to choose (52). That is to say, compared to individuals with no chronic disease, chronic patients are more likely to rely on mHealth both physiologically and psychologically due to their demands. For individuals who have less demands (e.g., individuals with no chronic disease), they show less stickiness to the service (53). For this reason, users with no chronic disease tend to change their usage intention due to their mutable evaluations of mHealth services. In this study context, expectation and confirmation correlate to users’ perception and evaluation of mHealth services, which are both likely to show stronger impacts on the change of use intention for individuals with no chronic disease. Thus, we put forward that:

*H7*: The relationship between expectation and rate of change in continuous use intention is stronger for individuals with no chronic disease.

*H8*: The relationship between confirmation and rate of change in continuous use intention is stronger for individuals with no chronic disease.

**Figure 1:**
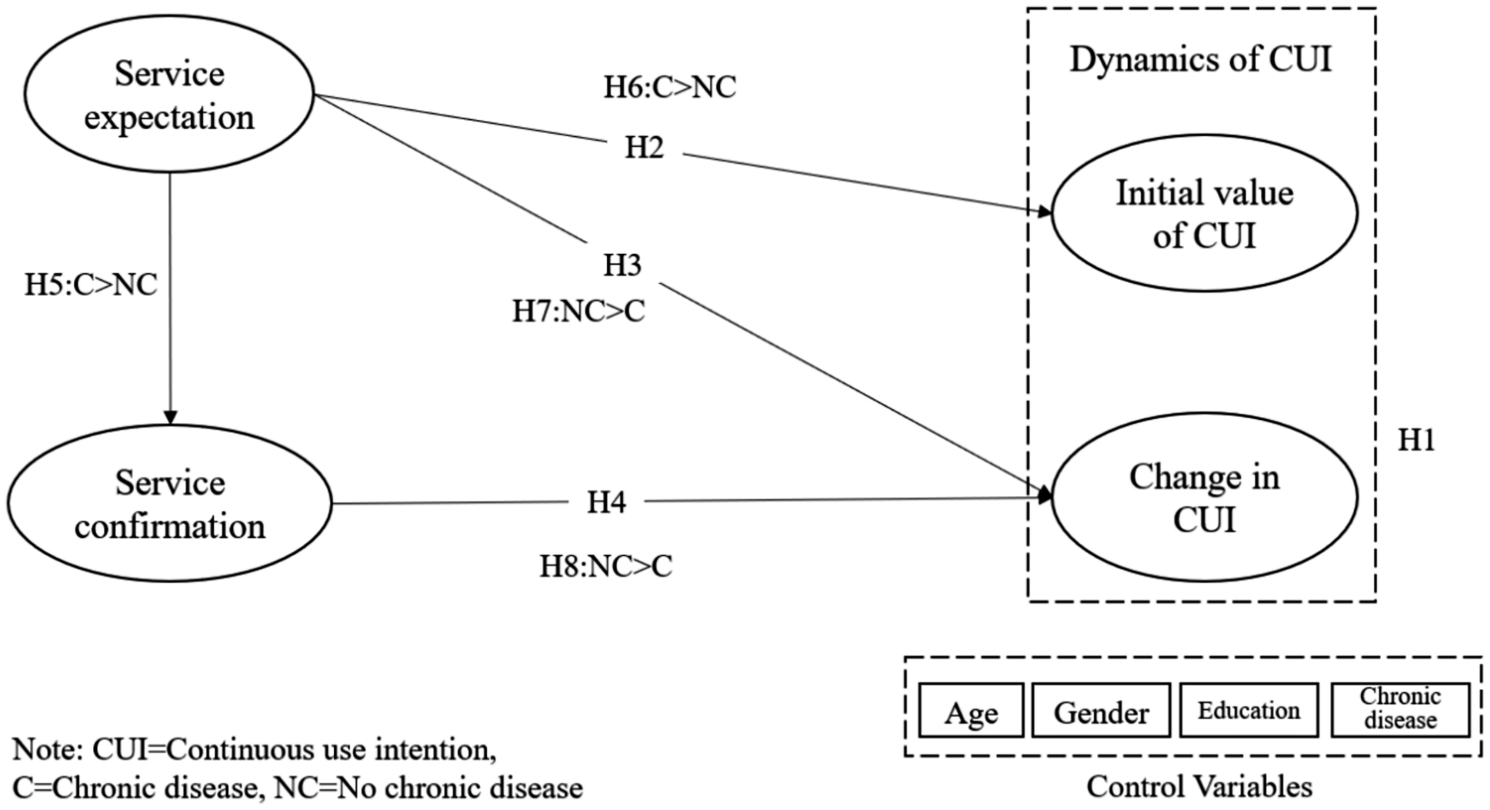
Research model.

To investigate the dynamics of mHealth services use intention from the ECM perspective and explain the role of chronic disease, this study employs a latent growth model. We build the research model based on ECM and empirically test the model with longitudinal survey data. The research model and hypothesis relationships are descripted in Figure 1.

## 3 Methods

### 3.1 Research setting

Most previous studies have examined expectation through asking respondents to recall their past expectations; however, memories of expectation are not accurate. Therefore, we collected data using multi-wave surveys which measured respondents’ perceptions in real time. To achieve this objective, we conducted this study in cooperation with a major hospital in Beijing, China. At the time, the hospital was launching an mHealth application that offered services such as health information education, online consultation, health index record and monitoring, and advice notification, similar to other mHealth services on the market. Both chronic patients and individuals with no chronic disease can use these services for self-health management. Individuals who came to the hospital’s health center for a physical examination were selected as target participants. These individuals are appropriate for this study since they did not come to the hospital for any specific treatment. Thus, their chronic disease status is random, and they are potential users of mHealth services.

### 3.2 Data collection

Participants who did not previously use the hospital’s mHealth application were recruited. We collected data at three time points (T1-T3) to explore the initial value and dynamics of users’ beliefs and their use intention. At the beginning, we introduced our research and the mHealth services to potential participants and invited them to join the study. Patients who agreed were immediately invited to download the application to their mobile phone and complete a survey (at time T1). The survey contained two parts: one part investigated their expectation and continuous use intention regarding the mHealth services, and the other collected their basic demographic information. Two months later, another survey link was sent to examine their confirmation and continuous use intention (at time T2). The last survey investigating users’ continuous use intention was disseminated after another 2 months (at time T3). Respondents participated in the research voluntarily and were able to quit the study anytime. All research activities had received institutional review board approval and followed approved institutional review board guidelines. Considering the high attrition rate in multi-time-point studies, we recruited 500 participants, of whom 462 respondents completed the first-round survey. For the second-round survey, 350 of the 462 participants responded to the questionnaire. Finally, 236 respondents completed all three surveys. To test the nonresponse bias, we compared the initial respondents and final valid respondents in terms of gender (t = −1.43, *p* > 0.1), age (t = 0.72, *p* > 0.1), education (t = −1.28, *p* > 0.1), and chronic disease (t = 0.31, *p* > 0.1). No significant difference was found across these characteristics, indicating nonresponse bias is not a concern. Of these 236 valid respondents, 53.4% were male and 44.1% had a chronic disease. The demographic information is shown in Table 1.

**Table 1:** Demographic statistics.

Variables	Category	Frequency	Percentage (%)
Gender	Male	126	53.4
	Female	110	46.6
Age	30 or younger	36	15.3
	31–40	84	35.6
	41–50	60	25.4
	51–60	38	16.1
	61 or older	18	7.6
Education	High school or lower	62	26.3
	College	44	18.6
	Undergraduate	78	33.1
	Postgraduate or higher	52	22.0
Chronic disease	Yes	104	44.1
	No	132	55.9

### 3.3 Measurements

All measurements were adapted from previous studies and modified to the mHealth context as shown in Table 2. We employed seven-point Likert scales ranging from “1 = strongly disagree” to “7 = strongly agree” to measure multiple items of latent constructs. Specifically, continuous use intention was measured with three items adapted from Bhattacherjee (31). Expectation was measured with three items adapted from Lin et al. (49) and confirmation was measured with items adapted from Bhattacherjee (31). Chronic disease was measured as a dummy variable based on respondents’ yes/no answers to the question “Are you suffering from one or more chronic diseases?” Variables such as age, gender, and education were measured and collected due to control purposes. Because the respondents are Chinese, we translated all items into Chinese with back-translation.

**Table 2:** Construct measures.

Construct	Item	Questions	Related research
Continuous use intention	CUI1	I intend to continue using the mHealth services rather than discontinue its use.	(31)
	CUI2	My intentions are to continue using the mHealth services than use any alternative means (traditional medical service).	
	CUI3	If I could, I would like to discontinue my use of the mHealth services (reverse coded).	
Service expectation	SE1	I expected the service quality of the mHealth services to be good.	(49)
	SE2	I expected the service quality of the mHealth services to be stable.	
	SE3	I expected the service quality of the mHealth services to be effective.	
Service confirmation	SC1	My experience with using the mHealth services was better than what I expected.	(31)
	SC2	The service level provided by the mHealth services was better than what I expected.	
	SC3	Overall, most of my expectations from using the mHealth services were confirmed.	

## 4 Results

This study aims to explore the effects of users’ expectation and confirmation of mHealth services on the change in their continuous use intention. The latent growth model approach is widely applied to examine how independent variables influence the change trajectory of dependent variables over time based on three or more longitudinal data sets (54). Thus, a latent growth model is employed for data analysis in this study. There are two latent factors involved in the latent growth model, namely intercept and slope. The intercept is a constant indicating the initial mean level of constructs measured at time T1, while the slope indicates the extent to which the mean value of the construct changes over time. In the latent growth model, the intercept and the slope were estimated as latent variables (55).

AMOS 24.0 is one of the various programs used to analyze the latent growth model and thus is employed to conduct data analysis in this study. Analyzing the latent growth involves two steps (56). The first step is to estimate the unconditional model which measures the dependent variable at three times. If the unconditional model is acceptable, the second step is conducted to estimate the conditional model, which evaluates how independent variables affect the slope of the dependent variable. Moreover, the bootstrapping approach was employed to validate moderating effects in the research model.

### 4.1 Unconditional latent growth model

There are two types of change trajectory in the unconditional latent growth model: one is the linear change growth (the slop loadings are labeled as 1,2,3…) and the other is non-specified cumulative growth (the first slope loading was fixed to 0 and the last was fixed to 1). To adopt the most suitable model in this study, we compared the fitness of these two models. The results showed that the non-specified cumulative growth model fit for the three stages of continuous use intention was (χ2 = 14.43, df = 2, *p* = 0.001, TLI = 0.505, NFI = 0.645, CFI = 0.670, and RMSEA = 0.163). In contrast, the linear model fit was (χ2 = 2.339, df = 3, *p* = 0.311, TLI = 0.987, NFI = 0.943, CFI = 0.991, and RMSEA = 0.027). Comparing the fit of these two models, we found that the linear growth model presented a higher-quality goodness-of-fit index and was superior to the non-specified cumulative growth model. Thus, we choose the linear change model as the latent growth model for analyzing continuous use intention in this study.

After adopting this model, we estimated the intercept (initial value) and the slope (rate of change) of continuous use intention as follows. The intercept of continuous use intention was 6.042 and statistically significant. The average of the slope of use intention was −0.273, indicating that use intention significantly decreases with a negative slope. This indicates that individuals’ intention to continue use mHealth services decreases significantly over time, supporting H1. Additionally, both the variance of intercept and of slope are significant, showing that individual differences exist in initial value and rate of change of use intention (see Table 3).

**Table 3:** Mean and variance analysis.

Mean and variance	Unstandardized estimate	S.E.
Intercept mean	6.042***	0.052
Slope mean	−0.273***	0.033
Intercept variance	−0.205***	0.065
Slope variance	0.143***	0.034

***p < 0.001.

### 4.2 Reliability and validity analysis

A confirmative factor analysis (CFA) was conducted to assess reliability and validity of construct measurement using collected data. The reliability can be estimated by evaluating composite reliability and Cronbach’s alpha. Table 4 showed that all composite reliabilities and Cronbach’s alpha values were higher than the suggested threshold value of 0.7 (57). Thus, all constructs showed a good reliability. To test convergent validity, we applied two criteria, factor loadings and average variance extracted (AVE), as suggested by Fornell and Larcker (57). Table 4 showed that all factor loadings were significant and greater than 0.7 and all AVEs were over 0.5, suggesting a good convergent validity of all constructs. To evaluate discriminant validity, we compared the square root of AVE for a given construct and the correlation coefficients between this construct and other constructs. As shown in Table 5, for each construct, the square root of AVE was greater than the correlation coefficients, suggesting the discriminant validity is acceptable.

**Table 4:** Reliability and convergent validity.

Variables and measurement items		Standardized factor loading	Cronbach’s alpha	Composite reliability	Average variance extracted
SE	SE1	0.943	0.904	0.940	0.840
	SE2	0.874			
	SE3	0.930			
SC	SC1	0.973	0.967	0.978	0.937
	SC2	0.960			
	SC3	0.971			
CUI [T1]	CUI1[T1]	0.919	0.923	0.942	0.844
	CUI2[T1]	0.929			
	CUI3[T1]	0.908			
CUI [T2]	CUI1[T2]	0.956	0.952	0.968	0.909
	CUI2[T2]	0.950			
	CUI3[T2]	0.954			
CUI [T3]	CUI1[T3]	0.825	0.870	0.911	0.773
	CUI2[T3]	0.917			
	CUI3[T3]	0.893			

SE, service expectation; SC, service confirmation; CU, continuous use intention.

**Table 5:** Discriminant validity.

Variables and measurement items	SE	SC	CUI [T1]	CUI [T2]	CUI [T3]
SE	**0.919**				
SC	0.114	**0.968**			
CUI [T1]	0.396	0.040	**0.919**		
CUI [T2]	0.134	0.519	−0.005	**0.953**	
CUI [T3]	0.122	0.333	0.130	0.388	**0.879**

SE, service expectation; SC, service confirmation; CU, continuous use intention.

Bold values refer to the square roots of AVE.

### 4.3 Conditional latent growth model

**Figure 2:**
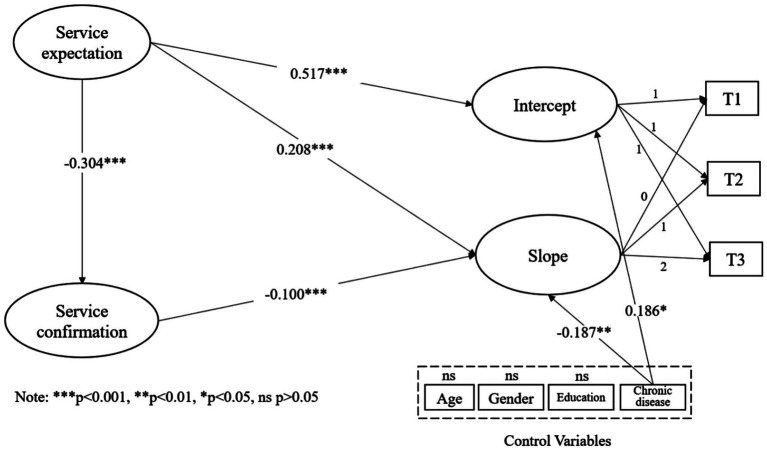
Model results.

To test our hypotheses, we constructed a conditional latent growth model including all independent and dependent variables such as expectation, confirmation, continuance intention intercept and continuance intention slope. The unstandardized loadings of continuance intention on all intercept factors were fixed at 1.0. The unstandardized loadings on the slope factors by T1, T2, and T3 were fixed at 0, 1, and 2 to set a linear trend for the change in continuance intention. The relationship between variables is shown in Figure 2.

First, the model fit was tested. The criteria for a good model fit includes chi-square/degrees of freedom (χ2/df) less than 5, NFI, TLI, and CFI greater than 0.9, and RMSEA less than 0.08 (58). The results showed a satisfying model fit for the indices: χ2 = 61.56, χ2/df = 1.466, NFI = 0.968, TLI = 0.980, CFI = 0.989, and RMSEA = 0.023. All fit indices satisfied the criteria.

Next, we examined how expectation and confirmation influence the intercept (initial value) and the slope (rate of change). We found that expectation positively predicted the intercept of continuance intention (β = 0.517, *t* = 6.702, *p* < 0.001) and positively related to the slope as well (β = 0.208, *t* = 3.642, *p* < 0.001), supporting H2 and H3. In terms of the effect of confirmation on the slope, the result showed a significant negative effect (β = −0.100, *t* = 4.785, *p* < 0.001), lending support to H4. We also found that expectation significantly decreased confirmation (β = −0.304, *t* = 3.420, *p* = 0.001). In terms of control variables, chronic disease was found to positively affect the intercept (β = 0.186, *t* = 2.169, *p* < 0.05) and negatively affect the slope (β = −0.187, *t* = −2.740, *p* < 0.01). Figure 2 shows the conditional latent growth model results.

To assess the moderating role of chronic disease, we manipulated a multi-group invariance test through nested model comparisons to check whether the path coefficients for the chronic disease group and those for the no chronic disease group statistically differed from each other. Parameters of the unconstrained model (M0) were freely estimated, and equality constraints were imposed, particularly for the parameters of the nested models (M1-M4). In this test, the chi-square difference between these two models should indicate whether the equality constraint holds across different groups (59). Table 6 showed the differences between the chronic disease group and no disease chronic group in the path coefficients of expectation-confirmation (*p* < 0.001), expectation-intercept (*p* < 0.05), expectation-slope (*p* < 0.01), and confirmation-slope (*p* = 0.098). Based on the results, H5-H7 were supported but H8 was not supported.

**Table 6:** Nested model comparisons.

Model	Path	Coefficients of chronic disease/no chronic disease groups	∆*χ*^2^	∆df	*p-*Value	Hypothesis support
M0 (*χ*^2^ = 127.9, df = 72)						
M1	SE → SC	−0.447 / -0.291	12.7	1	0.000***	H5 Supported
M2	SE → Intercept	0.526 / 0.384	6.46	1	0.011*	H6 Supported
M3	SE → Slope	0.167 / 0.323	10.81	1	0.001**	H7 Supported
M4	SC → Slope	−0.102 / -0.107	2.74	1	0.098 ns	H8 Not supported

SE = service expectation, SC = service confirmation, ***p < 0.001, **p < 0.01, *p < 0.05, ns *p* > 0.05.

## 5 Discussion

### 5.1 Key findings

We examined the dynamics of users’ long-term use intentions regarding mHealth services. From the point view of ECM, we investigated the impacts of expectation and confirmation on the change in users’ intentions, as well as the role of chronic disease. A latent growth model was used to analyze longitudinal survey data, and most of the hypotheses were supported. Here are a few vital findings.

First, this study offers several insights derived from its first research objective. Through a longitudinal analysis, the research demonstrated how users’ intention to continue using mHealth services is dynamic and decreases over time with a negative slope. This result supports the notion that users’ beliefs and intentions change across time (11). While users may have high expectations of mHealth services and a high initial intention to use them, the intention is likely to decline in subsequent use. Thus, these findings seem to buttress the mechanisms observed in cognitive dissonance theory (60), which indicates that individuals’ behavior and cognition will adjust as time progresses to keep behavior and cognition in balance.

Second, in light of its second research objective, this study clarified the impacts of expectation and confirmation on the change in users’ intention to continue using mHealth services. Consistent with prior research (61), this study validates that expectation is positively related to initial continuous use intention. Additionally, the results indicate that expectation shows positive impacts on the rate of change in continuous use intention. That is to say, when users have high expectations for mHealth services, their intention to continue using decreases more quickly. Contrary to expectation, however, this study finds that confirmation is negatively related to intention change rate, indicating that for users who have high confirmation, their intention declines more slowly. These results are consistent with the mechanism of the ECM. According to a previous study, confirmation leads to higher satisfaction and relatively higher use intention (31) and thus will slow the decline in continuous use intention. However, high expectation will cause low confirmation (41), ultimately leading to a faster decline in continuous use intention.

Third, considering the third research objective, the results suggested that chronic disease plays a vital role in mHealth services use. Specifically, compared to users with no chronic disease, chronic patients have higher initial continuous intention and their continuous use intention declines more slowly. Moreover, the results indicated that expectation showed a stronger impact on initial use intention but showed a weaker impact on the rate of change in intention for users with chronic disease than for users without chronic disease. In regard to the impacts of confirmation on the rate of change in intention, no significant difference exists between the two user groups. This finding may indicate that confirmation is critical for users both with and without chronic disease. Furthermore, this study found that there is a difference between the two groups with regard to the impact of expectation on confirmation: expectation has significant negative impact on confirmation for users with chronic disease, but it has no significant impact for users with no chronic disease.

### 5.2 Theoretical implications

First, this study advances the understanding of continuous use, particularly in the mHealth services context, by accounting for time-based dynamics. Although plenty of extant studies have contributed to an understanding of the post-adoption and continuous use of mHealth (62), they have primarily examined the continuous use intention at one time point under the implicit assumption that this intention is static. However, since users’ experience with mHealth is dynamic, their attitude regarding the service may not be static, and continuous use intention may change during a long-term use process (34). For example, a user may initially have a high continuous intention but may then change their decision or even stop using the mHealth services after a period of time. Examining the change in continuous use intention helps us more deeply understand the sustainability of mHealth use from a whole process perspective. This study opens new theoretical views for research on the conceptualization of continuous mHealth services use. In other words, when researchers investigate the continuous use of mHealth, they are suggested to consider both users’ initial continuous intentions and the change in their subsequent intentions.

Second, this study extends the ECM to a new dynamic model through examining the effects of expectation and confirmation on continuous use dynamics. As previously mentioned, several research gaps exist in previous studies that draw upon the ECM to explain continuous use of technologies, including a failure to address the dynamic nature of continuous use intention and a joint consideration of the impacts of pre- and post-beliefs. In this regard, the current conceptualization of the ECM cannot sufficiently capture the concept of development and change in users’ intentions. Thus, we build a research model to investigate impacts of pre-use expectation and post-use confirmation on initial intention and changes of intention in multiple time windows. This research not only expands the ECM by involving changes of continuous use intention but reveals the different impacts of expectation and confirmation. Thus, this study advances in study of Bhattacherjee and Premkumar (17) and improves our understanding of ECM from a dynamic perspective. Moreover, this study also responds to the appeal of Lee et al. (11), who called for studies on the ECM to consider the dynamic nature of processes within information technology usage. The findings advocate that future research on ECM would be well served by paying more attention to both users’ pre- and post-beliefs and to the dynamics of their continuous use intentions.

Third, this study investigates the role of chronic disease in continuous use related to mHealth services, which has rarely been examined. Although prior studies have explored the moderating effect of perceived vulnerability to chronic disease on users’ beliefs and preferences regarding mHealth channels (22), none have empirically tested the role of chronic disease in long-term mHealth use. It is therefore necessary to diagnose the differences between users with chronic disease and users with no chronic disease in terms of the dynamics of their mHealth use intentions. The results of this study demonstrate that chronic disease leads to higher use intention and plays nuanced moderating roles in relationships between expectation, confirmation, and changes in use intention. These findings suggest that differences in users’ health status (e.g., chronic disease) should be studied alongside user’s beliefs on mHealth services. Furthermore, this research enriches the ECM in the mHealth context through introducing chronic disease as a contextual factor. The results support the viewpoint of Hong et al. (63), who stated that integrating contextual factors into research models is able to cause new explanations and elaborations of theoretical understanding.

Fourth, using a latent growth model, this study provides a methodological guidance for future research about the dynamics of user intentions and behaviors. Since it is well suited for measuring the dynamics of constructs, the method employed here can be used to examine longitudinal adoption and use of technologies and services. Unlike prior longitudinal studies, which investigated constructs in two time windows and analyzed them as cross-sectional without considering the time-based dynamics (64, 65), this study provides guidance for research investigating dynamics of constructs with multiple time windows and additional factors that may influence the dynamics. Furthermore, the statistical methodology inspires scholars to establish more dynamic models examining users’ beliefs and technology use, thus making a theoretical contribution to the information systems and management discipline.

### 5.3 Practical implications

This research shows insightful significance for practice regarding ways to increase continuous use and improve the sustainability of mHealth. First, the results indicate that mHealth service providers should recognize that mHealth use intention is not static and decreases over time. Users may show great passion for using mHealth services but lose their enthusiasm for it gradually. Given that the high dropout rate is one of the most serious threats to the sustainability of mHealth (66), mHealth service providers should invest more effort in retaining existing users in addition to attracting new ones. For example, mHealth service providers can regularly investigate users’ attitudes about the service and continually monitor their use behavior. Then, when the provider detects that a user’s continuous use intention is dropping and that the user may become inactive, they can take immediate steps to retain them, such as surveying their expectation and optimizing the service to improve their confirmation.

Second, mHealth managers should note that although high expectation leads to high initial continuous intention, it accelerates the decrease of intention in the subsequent use process. Moreover, expectation is negatively related to confirmation, while confirmation is beneficial to continuous use. Thus, managers may want to strike a balance between building up users’ expectations and forming confirmation. Especially in the advertising phase, setting very high expectations of mHealth services is not the best option. We recommend that mHealth managers shape users’ expectations appropriately at the initial stage and then subsequently pay closer attention to improving users’ confirmation. For instance, mHealth developers can update mHealth applications, provide some new functions, and optimize the interface to improve user experience and the users’ confirmation of the expected performance.

Third, mHealth service providers and designers should consider individual differences in the process of providing services. Results in this study indicate that individuals with chronic disease and those with no chronic disease engage differently with mHealth services in the long term. Specifically, users with no chronic disease are prone to cut back on the service, especially when their initial expectations are high. Given that the mHealth industry is shifting from health management to health enhancement, this study suggests mHealth service providers and designers to provide personalized services for different users. For example, specific artifact designs and retention strategies can be aligned with individual differences.

### 5.4 Limitations and future research

We acknowledge that there are some limitations to be addressed in future research. First, the data were collected from users of a single mHealth platform in China. Since people living in different cultural contexts may have different experiences of and beliefs about mHealth services, the generalizability of this study’s findings for other populations remains to be explored in the future. Second, since the study mainly focused on the impacts of pre-adoption belief (e.g., expectation) and post-adoption belief (e.g., confirmation), only the direct effects of these two factors and the moderating role of chronic disease were examined. Future research could incorporate more elements; for instance, it could investigate other contextual factors in the research model. Third, this study was conducted in three time-windows and confirmation was examined in the second time window. We admit that there is a possibility that confirmation will change across time and we failed to identify the change. Future research could try to increase the number of data collection time-windows (at least four times) to measure the change of confirmation during this longer process. Finally, we examined users’ intention to continue using mHealth services rather than actual use behaviors, which could be the target of future investigation.

## 6 Conclusion

This study investigates the dynamics of continuous use of mHealth services. Building a latent growth model with a longitudinal analysis, this study identifies the change trajectory of users’ continuous use intention. Moreover, drawing upon the ECM, this study examines the impacts of expectation and confirmation on changes in continuous use. The results indicate that expectation is positively related to both initial use and the long-term changes in use, while confirmation is negatively related to changes in use. The analysis also shows that there are individual differences in the dynamics of continuous use as well as in the relationships between expectation, confirmation, and changes in use. The findings theoretically contribute to understanding the dynamic aspect of users’ continuous use of mHealth services and expanding the ECM through introducing chronic disease as a moderator. This study also provides guidance for practitioners, suggesting that they should pay more attention to retaining current mHealth services users and developing strategies to facilitate their continuous use.

## Data Availability

The original contributions presented in the study are included in the article/supplementary material, further inquiries can be directed to the corresponding author.
